# Exploring the divide between dental clinicians and academics for more inclusive partnerships: perspectives on building a research network

**DOI:** 10.1186/s12903-025-06437-w

**Published:** 2025-07-06

**Authors:** Maria Zych, Carilynne Yarascavitch, Amir Azarpazhooh, Joanna Sale

**Affiliations:** 1 Instruction and Liaison Librarian, Dentistry Library, University of Toronto Libraries, Toronto, Canada; 2 Dental Anesthesiology, Faculty of Dentistry, Dentist-Anesthesiologist, Department of Dental and Maxillofacial Sciences, University of Toronto, Sunnybrook Hospital, Toronto, Canada; 3 Endodontics, Faculty of Dentistry, Clinician Scientist, Lunenfeld-Tanenbaum Research Institute, University of Toronto, Mount Sinai Hospital, Toronto, Canada; 4 Li Ka Shing Knowledge Institute, St. Michael’s Hospital, Unity Health Toronto, Toronto, Canada; 5 Institute of Health Policy Management and Evaluation (IHPME), Dalla Lana School of Public Health, University of Toronto, Toronto, Canada; 6 Department of Surgery, Temerty Faculty of Medicine, University of Toronto, Toronto, Canada

## Abstract

**Introduction:**

Dentists experience barriers of applying knowledge at point of care. In recent years, integrated knowledge translation (IKT) efforts, such as dental practice research networks, have been established in the US and other countries to overcome the potential barriers to dental clinicians being involved in research. In Canada, there is no formal network. The objective of this study was to explore the perceptions of dental clinicians and academics in building a network in Ontario that would encourage research collaboration.

**Methods:**

Semi-structured interviews were conducted to collect data from dental clinicians and academics in Ontario dental schools. Purposeful sampling was used to recruit participants from the Faculty of Dentistry at the University of Toronto and Schulich School of Dentistry at Western University. Interviews were conducted via Zoom meetings between July and November 2023. Interviews were recorded, de-identified, transcribed, and analyzed using thematic analysis. Strategies to promote rigour were applied throughout the research process.

**Results:**

Twenty-six interviews were conducted (twelve dental clinicians and fourteen academics). Most participants described a divide impacting many aspects of oral health research. This divide appeared to be originating from historical cultural norms rooted in the development of organized dentistry in Ontario, current financial strains faced by dentists, and misalignment of values that resulted in miscommunications between dental clinicians and academics. Despite the divide, there appeared to be a strong desire among participants to “bridge the divide”. Suggestions included starting a small network of interested individuals, enforcing existing collaborations within the universities, identifying champions in organized dentistry to advocate for establishing a formal network in Ontario, and exploring opportunities to support such a network in Canada.

**Discussion:**

Despite the divide between dental clinicians and academics in oral health research, there appeared to be a strong desire to bridge the divide. This study is novel in that it reports dental clinicians’ and academics’ perceptions and experiences with IKT partnerships in dentistry. We offer recommendations for oral health professional leaders to build a new network.

**Supplementary Information:**

The online version contains supplementary material available at 10.1186/s12903-025-06437-w.

## Background

Evidence-based dentistry (EBD) promotes dialogue between dental practitioners and oral health researchers so that best available evidence is used to inform clinical decision-making [1]. Although EBD courses are effective in impacting the knowledge and attitudes of dental students [2], barriers still exist in applying EBD in practice [3–6]. Morris et al.’s 2011 article describing the 17-year lag between knowledge creation and its use [7] is still recognized today as an issue [6]. In dentistry, the lag between knowledge creation and use is widely known [8, 9]. Dentists continue to experience barriers of applying knowledge at point of care [9, 10]. The tool Caries Management System, outlines recommendations for treatment of caries and risk assessment in adults [11], children and adolescents [12]. Despite availability of this tool, a survey of dentists in the American Dental Practice-Based Research Network showed that only 69% of dental practitioners reported performing this assessment on their patients [13]. A 2014 cross-sectional study in the US asked dentists how they would treat patients in 12 different clinical scenarios and compared the answers to care recommendations based in the current evidence [14]. Overall, only about 62% of recommendations were followed by practicing dentists [14]. Lack of evidence-based practice is also evident in a 2016 survey of dentists in Japan for dental sealant application in dental caries prevention [15]. In Canada, there are also reports of barriers to applying periodontal research evidence into practice, including insufficient time, difficulty staying current, lack of clarity in research [16]. In addition, there are reports of incorrect implementation of practice guidelines for prescribing antibiotic prophylaxis for dental procedures on patients at risk of infective endocarditis [17–21].

Knowledge translation refers to the uptake of knowledge by practitioners at the point of care, and it is used to describe the effort to reduce the gap between knowledge creation and its application [22]. There are several approaches to knowledge translation. Integrated knowledge translation (IKT) refers to an approach where knowledge users (those who use evidence or are impacted by evidence, such as health care providers, patients, caregivers, parent groups, etc.) are included as partners for knowledge co-production to improve knowledge uptake [23, 24]. In this model, researchers and knowledge users collaborate as partners in knowledge co-production with the goal to increase the uptake of knowledge into practice [24, 25]. IKT is unique from other co-production of knowledge approaches in that knowledge is generated by a true partnership between researchers and knowledge users, rather than simple engagement of knowledge users [26]. The benefits of IKT are that knowledge users are more involved in the process of knowledge creation and therefore more likely to apply it; it generates knowledge that is better informed to practice and improves understanding of the outcomes from both parties [27]. In spite of its benefits, this type of knowledge co-production remains underused [28]. Barriers to this approach include lack of time, lack of organizational support to be involved in and implement research, and lack of feedback about the outcomes [29].

A recent literature review by Randall suggested integrating clinicians in oral and craniofacial research to make evidence more applicable at point of care [10]. There are some initiatives already in the US and other countries that aim to increase the collaboration between dental clinicians and academics in oral health research for co-production of knowledge. IKT partnerships, sometimes referred to as practice-based research networks, are beneficial to the uptake of research evidence at the point of care [30, 31]. In recent years, dental practice based research networks (PBRNs) have been established to overcome potential barriers for dental clinicians to participating in research studies [32].

The relationships between dental clinicians and academics in oral health research in the context of knowledge co-production is not well explored [9]. A 2019 qualitative study involving Canadian researchers and knowledge users suggested that a forum for sharing ideas and connecting would help initiate partnerships [33]. There is no known forum where dental clinicians and academics connect to conduct IKT research in Canada. The purpose of this study was to describe the perceptions of creating a forum or network in Ontario for IKT partnerships between dental clinicians and academics. In particular, this study aimed to answer the following research questions: (1) *What are the perceptions of clinicians and academics to engaging in IKT partnerships?* (2) *what are desired features of a dental clinician-academic IKT network?* The results could be applicable to the greater Canadian landscape, given the similarities in funding opportunities across provinces.

## Methods

Qualitative research is appropriate for examining the preferences and perceptions of partnerships between dental clinicians and academics in oral health research, as we aimed to explore preferences and insights on creating a network for easier collaboration. We used a constructivist approach, which is consistent with qualitative description [34, 35], in that our aim was to develop knowledge that was constructed through interactions between the research and the research participants, and allows the researchers to construct meaning from data [36, 37]. Semi-structured interviews were conducted to collect data from clinicians and academics in Ontario dental schools. Semi-structured interviews provide flexibility for participants to speak openly about their experience while keeping the conversation aligned with the study goals [38]. A literature search on the topic of IKT and dentistry was conducted using the databases MEDLINE, EMBASE, Web of Science and Scopus. The interview guide (see Additional File 1) aimed to fill some of the knowledge gaps found in the literature review. Topics covered in the interviews included asking about past opportunities to take part in partnerships, existing networks and partnerships between dental clinicians and oral health researchers in Canada, and preferences for a research network [13–16, 18–21, 39–45]. Questions in the interview guide were reviewed and revised by the research team (MZ, CY, JS, AA) [46]. We adhered to the Standards for Reporting Qualitative Research for critical appraisal of our methods and reporting our results [47]. University of Toronto Research Ethics Board (which adheres to the Declaration of Helsinki) approval was received for this study (Protocol # 44545). Purposeful sampling was used to recruit participants [48, 49]. The recruitment strategy included dental clinicians and academics in the two Ontario dental schools: Faculty of Dentistry, University of Toronto (UofT Dentistry); and Schulich Dentistry, Western University. We defined dental clinicians as dentists and dental specialists who taught in the schools’ dental clinics or lectured in didactic courses and were active in private practice. We defined academics as those who currently or recently held faculty positions, supervised research, and/or held senior administrative roles at the dental schools. To recruit clinicians, an email was distributed to the listserv of all Clinical Instructors at both universities. To recruit academics, an invitation was distributed to the listserv via the research offices at both universities. Guest et al. (2006) estimated a sample of twelve interview participants per group to reach saturation, when the goal is to determine how two or more groups differ along a given dimension [50]. We aimed to recruit 12 to 15 of each group: dental clinicians and academics, to make the participant pool equal [21]. Sample size was determined by a number of factors, such as the scope of the study being small (including only two dental schools in Ontario), the topic was very focused, and that we would recruit information rich participants [51]. Along with the invitation email was a link to a demographics form on MS Office 365, collecting data on years of experience, sex, degrees and university of Doctor of Dental Surgery. Due to the lack of access to the listservs by the interviewer, the number of recipients of the email is not known. We interviewed everyone who responded. Informed consent was obtained from participants prior to the interviews. Their rights to participate as well as their right to withdraw from the study at any time were outlined in the letter, which was signed by all participants.

Interviews were conducted by a team member with experience in qualitative research on Zoom with no other person present other than the participant and interviewer between July and November 2023. The interviewer (MZ) was known to some participants prior to participation. Audio files were recorded and transcribed. Field notes taken served as a method to reflect on the content of the transcripts. We analyzed the data using the six steps of thematic analysis [36, 37]. Step one included familiarizing ourselves with data by spending time reading the transcripts. In step two transcripts were coded using NVivo12 software to organize and sort the data [52]. In order to promote a comprehensive approach to coding, three transcripts were coded by team members (MZ, JS) independently, then discussed to develop a codebook for the remaining transcripts. While acknowledging our familiarity with the literature on the topic of IKT, we used primarily an inductive approach to data coding and analysis which allowed us to derive codes and themes from the content of the data collected [53]. Step three included noting common themes independently. In step four the resulting themes were discussed iteratively in two separate Zoom meetings and four emails (MZ, JS, CY) and in step five we agreed upon the naming of the themes. Finally, step six, was generating the manuscript with resulting themes. We used several strategies to promote rigour, including developing an interview guide informed in part from the literature review, discussing themes in an iterative process by multiple researchers, supporting themes with quotations from participants, and reporting negative cases [54].

## Results

Twenty-six interviews were conducted from July to November 2023. The participants included twelve dental clinicians and fourteen academics. Out of the twelve dental clinicians interviewed, there were five practicing dentists or dental specialists with a current or past appointment of Clinical Instructor at one of the dental schools (no didactic teaching role); and seven dentists or dental specialists who are involved in teaching or course delivery within the DDS and/or Graduate curriculum. The fourteen academics were all appointed faculty members. Ten were appointed to the supervise their own and graduate research, and four were senior administrators or emeriti, whom did not currently supervise students. Precautions were applied in order to de-identify the transcripts, such as excluding the reporting of some demographic information. Participants were affiliated with either the University of Toronto or Western University. Each section below provides representative quotes, however, Additional File 2 has a comprehensive report of supporting quotes for each theme. Quotations identified as “C” refer to clinician participants and “A” refer to academic participants. Our analysis identified two major themes: *Across the Divide* and *Bridging the Divide*. Several sub-themes were also identified for each major theme and are presented below.

### Across the divide

Across the Divide included participants’ perspectives of the cultural division between dental clinicians and academics, and the resulting challenges to participate in research. When asked about past experiences with partnerships between clinicians and academics, our participants had very little to share. Only five had examples, with four of them admitting that it was not a true partnership (see Additional File 2).

#### Perception of the ‘other’

Participants shared perceptions of one group versus the other as being too busy to collaborate in IKT partnerships. Academics expressed their own busy commitments compared to dental clinicians, as a hurdle to collaborations. Some felt that their schedule was busier than dental clinicians’ and it would be difficult to make time for organizing IKT projects:

“This will sound wrong. So I’m not saying that academics are busier than private practitioners. But I think academics have the disadvantage of having more on their plates in the sense that you know, when you close the door, you are still not rid of your work…I know this because I also work in private practice…” [A4].

One academic reported that dental clinicians would not be interested in research due to lifestyle choice:

“The difficulty becomes I think, in our profession is that many, many people pick the profession for lifestyle. So when they get out, they’re okay to go to work, but they’re not interested in doing much beyond their work hours” [A6].

“The system is set up as such that you will not achieve that goal [the Ontario network]. So effectively we got…on one side a bunch of research scientists or basic scientists of whatever you want to call them, said to do research and in many cases do not understand a clinical problem, and do their research in their own corner…on the other side, you’ve got a clinician…would like to do research, but they don’t have a ton of resources or experience to do that. So it’s a very difficult equation” [A7].

On the other hand, some dental clinicians thought that academics may not be interested in collaborating with them:

“We’d also have to find out what the appetite is from the research end of things. Maybe they don’t want to. Maybe they look at it [and say] this is going to be more complicated…when we get more people involved.” [C3].

“if they [academics] have an open mind and they are open to discussion, then, I find that ego is not an issue here. And that’s really important in collaboration, because my experience has been that academics have an ego… [they] think they’re better than the clinicians…you can’t discount the clinical knowledge that an experienced practitioner has.” [C8].

Participants from both groups said it may be challenging to involve dental clinicians in research due to their lack of training or knowledge about the research cycle, forming research questions, and taking part in research activities, such as data analysis and interpretation. The perception that dental clinicians needed more training in research was expressed.

#### Perceived lack of respect

Participants from both groups discussed occasions when they felt disrespected because of their clinical research focus or their lack of research experience. According to participants the lack of respect was a barrier to collaboration and to obtaining funding required for clinical research:

“Unfortunately, it’s undermined [clinical research]. Inadvertently, I’m not saying it’s deliberate. But those who are reviewing our work. They’re not seeing how relevant it is to the public, to our patients. They’re not seeing the relevance of answering these clinical questions….It is so important that people recognize clinical research…so where is the clinical research being done? And we don’t have the money and neither do we have the resources to apply for a grant” [C8].

Some participants reported personal experiences where they felt clinical research caused a further division between the group of academics who were more interested in clinical areas than the basic research areas:

“[lack of collaboration] reflects the genuine lack of respect amongst those that have chosen to work more towards one side of the fence. Whereas there should be no fence here. But you know the fence exists and people think of themselves as either highly academically oriented, therefore more proficient…than those that are more in clinical, or seem to be at the other side of the fence, and that fence needs to be broken down….” [A9].

#### Physical and social barriers to collaboration

Participants spoke about how common physical spaces for discussions was an impediment to learning about research conducted at the schools or about each other’s research interests:

“Because I see a lot of people in the elevator, and I see them press the button for the [research area], and I have no idea who they are. Now you meet some of them at [annual event for research], which is something nice… where we bring the research into the mainstream where it’s actually oh, people in the rest of the building can go see what’s going on. But it’s not that common…” [C3].

“So I know there’s a lot of clinicians here, in our faculty, who really just work in the clinics. They’re on their feet all day, they don’t leave the [clinical area]. They’ve never come up. I’ll invite them up to the [research area] and they go “There’s a [research area]?” I’m going, yeah, there’s a [research area], that’s where all the research is, you’ve never been? So yeah, there is really [this divide].” [A5].

Dental clinicians reported feeling that more information should be shared about research projects, including sharing the outcomes and how they are relevant for practice, and to increase transparency on how and why the research questions were created:

“I know that they send us emails. I get emails all the time for research rounds or this or that…they [are at] very inconvenient times. It’s usually your lunch hour, and I’m not even at the faculty, I’m seeing patients, which is not my lunch hour there. So you know, maybe providing a little bit more variety on the times that they’re available. Would capture different people and not the same people all the time.” [C9].

Some participants implied that cultural norms had to change to make opportunities for participation more equal and welcoming to diversity and inclusivity:

“So it’s like an old boys club which is very difficult to penetrate. They play among themselves. They don’t want to involve any new ideas….So, having an open platform circulating, who wants to participate in this, you know, come forward….[Currently, [when there is an announcement of a new research project, the recruitment strategy is: ] this is open, now [it’s] closed, because we already filled the quorum with 5 of our good friends who [have] already been working on this. That’s what has been happening, and that’s not a good thing. What happens once they retire?…How will we get the legion of good data? And it has to be like, it’s an open platform, not on a closed group of people deciding on who to be in their study, who is going to contribute? Who’s going to get the money? It all boils down to that.” [A13].

### Bridging the divide

*“Bridging the Divide”* included perceptions and suggestions on how a network could improve communication between the two groups, including incentives to stimulate participation and possible funding opportunities to build and support a network. There appeared to be a strong drive among participants to “bridge the divide”.

Participants reported that there was a lack of networks for collaborations and lack of funding structures for dentistry, which they perceived to not be present in other fields, such as medicine:

“In medicine, you know, half the doctors at [hospital] are clinician scientists. You know they have done PhDs, they’re supported in their clinical research endeavors…graduate students, to get their specialty license, have to do research.” [A10].

#### New collaborative research network

Participants expressed a need to remove the barriers for collaborations between clinical and basic research in dentistry. The idea of a network was introduced to our participants and twenty-three participant agreed (ten out of twelve dental clinicians and thirteen out of fourteen academics) that a network to bring collaboration between dental clinicians and academics would be beneficial to co-production of knowledge in dentistry. Our participants were aware of the difficulty in planning and maintaining a network and that it would be challenging to form:

“I think it’s a great idea. I think you have some logistical hurdles to go through, but they’re not insurmountable. I think they’re totally doable. And I think, for the dental community, it would be really good. It brings us together just a little bit more, and gets everybody interested” [C1].

#### Using existing channels for a networks

Participants were asked about their professional or social networks, and if any of these were/could be used to connect with each other for IKT partnerships. Most participants expressed being closely connected with the Ontario Dental Association and the Canadian Dental Association, specialty associations and groups within Canada and beyond, and the Royal College of Dental Surgeons of Ontario. Communication seemed to flow well in the professional associations in terms of email communications, organization of events, and opportunities for continuing education points. Participants suggested that these associations could be used as a tool to communicate about research opportunities, but should not support a research network due to lack of resources. The network would have to be a stand-alone entity:

“Well, I guess the challenge would be…it becomes an administrative nightmare, you know, sort of organizing all this. So there would have to be sort of a formalization of almost like a not a corporation, but an entity, that would oversee the administration of something like this” [A12].

It was important to participants that if a network for collaboration was formed, the following groups should be invited at the discussions from the very start: dentists, dental specialists, academics and senior administrators from both schools, an identified professional association in Ontario, patient and caregiver groups, champions in organized dentistry, and funders, such as government agencies and private industry.

#### Administrative and funding support for clinical research

Clinical research was valuable to all our participants regardless of which group they identified with. Actions to show support for clinical research could include hiring personnel, such as a clinical research coordinator, or someone who could lead the projects by organizing team meetings, keeping timelines, and ensuring clinical standards were followed. This role would act as a point of contact for team members and take on project management responsibilities:

“Have one team leader that connects between the researchers and the clinicians, right? And if there are any questions, other than on the meeting…that they can email them [to the person] [C1].

“So I mean the person has to be properly compensated, right? It’s not going to be like $20,000 a year. It has to be something that’s appealing to someone as a career…that involves benefits and vacation pay and sick days…” [A3].

“…administrative people that should be helping us or could be helping us, to write grants. [Because] a lot of the times. We have the ideas. We just don’t have time to sit down to write the grants, right?” [A12].

Participants expressed that funding was required to support a network. Both private and public support should be explored in order for collaborations to have the finances for projects:

“It would have to be funded by the Government.” [C10].

“I think we would have to explore other types of [financial support], maybe industry…depending on what we are testing or evaluating right? If it’s extremely translational… Oh I think an MOU [Memorandum of Understanding] has to be very strong…because at the end…the data belongs to both, and a huge agreement has to be obviously established, so that the data is protected.” [A8].

#### Commitment and incentives

Dental clinicians admitted to being busy, however, they were willing to “carve” out time for research, if it was something that interested them personally or benefitted the profession or patients. They said they were willing to give up personal time after hours or even a business day out of their practice to be involved in research projects:

“I think that probably the single biggest barrier is just not knowing how to take the first step and get involved. I think, there’s a lot of great academics and researchers who can be sort of the brains of these projects. I’m happy to be a worker bee, collect data…I guess, not knowing the first step to take to say, Okay, hey, we’re interested. These are the resources we have that we’re willing to share with the academic community. How can we help?” [C6].

Participants said that dental clinicians may be incentivized to participate in collaborations by being offered continuing education points, authorship, knowledge acquisition, monetary incentives, and promotion of private practice as being connected with an academic institution. Academics reported that if a topic was of interest to them, they would be willing to participate in a research project without any additional incentives.

#### Expanding knowledge of dentistry

We asked participants if they had research project ideas that could benefit from more collaboration between dental clinicians and academics. Project ideas were summarized in Table 1. Some topics included patient-centered care, collecting public health related data so to better understand and describe the value of dental specialties on the overall healthcare system, data on the value of regular preventive care, and exploring minimally invasive procedures over invasive and costly procedures.

**Table 1:** Priority topics for network

Priority Topics	Participant Responses
Patient outcomes related topics	C2: “I think patient-centered care. I think that’s very important right now. And things happening and with dentistry being based on a clinician’s perspective on a business perspective, too. And when I think post COVID that things are moving away from patient-centered care. So I think that’s very important, like we do a lot of it as part of academia and teaching the students, but having something focusing on that and trying to reinstate that in private practice, too. I think, and having some ground rules for that, I think that’s something that should be worked on.” C4: Prospective [studies], like in private practice, I think prospect. It would be amazing. I mean, because we have so much. We have such great follow up for years. And our patients come back. C10: Conservative management of patients over a lifetime and the value of regular preventive care C11: The other aspect that I am trying to explore is coming from a clinical teaching. I am spending more and more time in the clinics working with students directly. I am seeing a very specific type of patients at the dental school with their demographics and their dental status at the dental school. These are the patients I don’t see a lot in private practice…really, they can only afford the most basic care which often involves extractions and immediate dentures. The more I work on these cases with the students the more I realize this is a whole new art in dentistry. We have to devise more effective ways, because really the dentitions are very challenging. Patients are very challenging A3: Minimally invasive procedures…the least invasive procedure, with the least risk. A11: I mean, the end product is really something that will be translated into patient care. I think that’s the whole point of research
Public health/public policy related topics	C6: What is our specialty doing? How many of us are in private practice versus academic settings? Part-time versus full-time? How many patients we see per year? How many patients we keep from seeing the OR? C10:…something that has the potential to move the dial on dental care. Especially in conjunction with this new kind of dental care program would be, you know, what is it that people need over a lifetime to keep their mouths healthy? A2: I think we’re continuously trying to establish in our specialty, the value of it. In terms of you know, economical opportunity to provide care in an economic way.
Clinical trials	C6: I think it would be really interesting to be involved with dental products and techniques
Understanding dentistry in different geographic areas	C12: Why many dentists in private practice in the inner city don’t want to see people who are homeless, and what we could do to make their offices more welcoming to people who are homeless A4: City versus rural, or rural versus West Ontario versus East Ontario. So the more people we could theoretically [have] involved, the better picture we would get as to where dentistry stands today…but we definitely could benefit from having more research to actually map our profession, how we are doing in general and what the regional differences are, of which, we have really no clue about.
Dental curriculum	A1: I suppose it would be helpful to know where they [dentists] perceive themselves to be lacking in knowledge. Because then, you could target a curriculum in the school that would try to address that.

#### Negative cases: an Ontario network not necessary

There were a few instances where participants said a network was not needed in Ontario because they were able to connect for research projects via an existing personal network within the dental schools which they created by their own efforts, or via existing journal clubs and networks in their specialty. These responses did not fit with the main themes, however, they are reported below in Table 2 for transparency.

**Table 2:** Network not needed or is not possible

Theme	Participant Responses
Connecting via existing channels	C3: “And normally, if I want to be involved with something, I can search it out. I can figure out, I can talk to people, and I can walk up to the [research area] and say, I want to get involved with some research. And I’m sure they would say “Yeah, we’ll find something”. So collaborating, given that I’m on the inside, I guess you could say, collaboration is really much easier than if I’m on the outside.” [C3]
	A4: “And for instance, I go to the specifically the [study club]. Both academics and private practitioners are there…it’s not a conference, it’s more like a study club kind of thing. But I think it is related. So I think that’s a good forum. Sometimes, you know, I think study clubs are primarily what would be appropriate for this.”
	A13: “So right now, I do hold study clubs…so I can talk to general dentists…. we need to have continuing points, and I participate in these lectures, where I try to connect with the general dentist, at the same time”.
Ontario network would be nice, but Ontario (or Canada) is too small	A6: “I’m not sure Ontario is the a large enough population.”
	A7: “There is still that sort of separation in [Canadian] dentistry between being a health care provider and being a business person….by business [person], I mean, I need to see X number of patients in my chair every day, because XYZ and so on….So there is a business side of dentistry that detracts a little bit, a lot I would say, from the innovation, from being able to spend time with the basic researcher, because there is money to be made”
	C5: “Dentistry is not a big enough field for that, and it’s just barely a big enough field for that….and there aren’t very many [specialists] in the country.” C5: “I think there are a number of barriers to that [creating a network]. A lot of it is business related, some of it is regulatory related, and the final piece is…would it make a difference, anyway? Like, if you made this new thing, is it so much better that you’re going to throw away the old thing in order to use this new [one]?”
	A6: “So I think it would have to be a bigger catchment area than Ontario to get the critical mass of people that you would need to make itself sustained. Now, whether that’s national or international…”
	A14: “The ADEA [American Dental Education Association]…it’s not just US, it’s US and Canada…the ADEA in the US is huge, and they have incredible resources that allows them to publish and do studies. They can’t do that in Canada, We are just too small. We don’t have the resources to fund that, to pursue it. It’s a matter of size to be honest” [A14].
	A12: “Well, I guess the challenge would be…it becomes an administrative nightmare, you know, sort of organizing all this. So there would have to be sort of a formalization of almost like a not a corporation, but an entity, that would oversee the administration of something like this”.

## Discussion

In this study, there was a perceived divide between dental clinicians and academics in oral health research. However, we also demonstrated that a network to support IKT initiatives appeared to be desired by the clinicians and academics interviewed. The divide preventing collaboration between clinical and basic research should be explored further. There have been reports about the challenges in the development of organized dentistry in Ontario, in particular the struggle for dentistry to be recognized by academia as a health profession [55]. A scoping review of 13 studies of IKT in healthcare, found that there are similar sentiments between clinicians and academics in other fields [27]. Some of the barriers to partnerships reported are misalignment of goals, failure to overcome differences and bridge boundaries, and lack of engagement of all stakeholders [27]. The lack of respect and undermining of clinical research has also been described in the literature of other disciplines. A review from 2020 analyzing principles, strategies, outcomes and impacts of research partnerships, found that relationship building between collaborators is crucial to research partnership success [56]. Values, such as trust, respect, dignity and transparency have to be aligned and communicated for partnerships to succeed [56, 57]. In addition, there are similar perceptions of building mutual respect and trust when engaging stakeholders in research collaborations [27]. This view is shared across disciplines, where the commitment of both parties (knowledge users/clinicians and academics/researchers) is crucial in the success of partnerships [26, 58].

Our participants reported a desire for a shared physical space where discussions could take place. This is not unique to dentistry. Similar views have been reported about physical spaces supporting partnership collaborations in other fields [59–61].

The undermining and underfunding of clinical research is also not unique to dentistry. However, it is unclear from this study whether dentistry is subject to more underfunding of clinical research in Canada than other disciplines [62]. Further investigation is required to explore if existing networks can be transformed to be more inclusive of research collaborations, or if strategic planning of a new network should be pursued.

Should oral health professional leaders, such as dental school administrators, professional organization leaders, or members of the dentistry community decide to explore building a network for collaborations, we propose several recommendations. First, the network should start locally and improve collaborations between clinicians and academics within the universities. Transforming physical spaces to allow for discussion opportunities, sharing of ideas, and the building of respect for each other would help. Second, the profession should call on champions from organized dentistry (leaders in the dental associations and dental schools) to initiate a conversation with the larger community about collaborative opportunities. Third, there needs to be an assessment of existing funding opportunities that support collaborations in Canada and promote these to oral health researchers. Fourth, we recommend that oral health professions invest in personnel to provide clinical research support for projects in order for the network to succeed and establish continuity. Fifth, existing or new opportunities should be reviewed with a diversity, inclusion and equity lens, to ensure that channels of participation are open, fair and equitable for everyone who is willing to participate in partnerships. Finally, dentistry needs to build a strong communications strategy to ensure that, in the long term, clinicians outside of the universities can participate. This would diversify and enrich data on access to care.

This study is novel in reporting Ontario dental clinicians’ and academics’ perceptions and experiences with IKT partnerships. Participants’ insights informed how a network can be created, or how existing networks can be adjusted to encourage collaboration, not just in Ontario, but the larger Canadian context. The limitations of the study were that insights were gathered from dental clinicians and academics who were connected to universities which excluded those who were not affiliated with the dental schools. In addition, oral health professionals, such as dental hygienists, dental technicians and dental assistants were not recruited in this study.

## Conclusion

Despite the divide between dental clinicians and academics in oral health research, there appeared to be a strong desire by participants to bridge this divide. Thus, this study is a starting point in the discussion to create a more collaborative and inclusive approach in oral health research in Ontario, and possibly nationally. Based on the interviews, we offer recommendations for dentistry leaders to build a new network.

## Electronic supplementary material

Below is the link to the electronic supplementary material.

Supplementary Material 1

Supplementary Material 2

## Data Availability

The anonymized transcripts generated are not publicly available. A summary of the transcript by theme is available in Additional File 2.
