# Supplementary material for: Exploring the divide between dental clinicians and academics for more inclusive partnerships: perspectives on building a research network
Source: BMC Oral Health. 2025 Jul 6;25:1122. doi: 10.1186/s12903-025-06437-w (PMC12232693; doi:10.1186/s12903-025-06437-w)
Supplement: Supplementary file 1 — Supplementary Material 1 [file 12903_2025_6437_MOESM1_ESM.docx]

**Additional File 1: Interview Guide**

QUESTIONS

**PHASE 1: INTRODUCTION/CONTEXT**

Can you tell me a little about yourself?

What are your clinical or academic qualifications?

What are your research interests?

**PHASE 2 EXPERIENCES WITH RESEARCH PARTNERSHIPS**

**Have you ever considered/tried partnering with a researcher/clinician to explore ideas? (IF NO, MOVE TO PHASE 3: SOCIAL AND PROFESSIONAL NETWORKS)**

Prompts

Who was in the partnership, and what was your role?

Who initiated the partnership? Tell me a bit more about the project.

How was it initiated (i.e. email, was there a launch meeting, etc.)?

Who led the team?

How long was the partnership? Tell me a bit more about the project

What was the outcome of the study? Tell me a bit more about the project

Would you consider the partnership successful? Why or why not?

**(If many partnerships, can you consider directing to the most recent, as this may be remembered best).**

- What was the most recent research partnership?
- What was the most memorable one?
- What was most successful and why?
- Were any unsuccessful and why?

**What training or skills have you developed that you can use/bring in a research partnership?**

**PHASE 3: SOCIAL AND PROFESSIONAL NETWORKS**

**Are there any current professional networks that you use or know of that may be useful to connect researchers and clinicians?**

Prompts

- Would you consider yourself part of a research network? If NO: Is this something that interests you? Why or why not?
- What are your social or professional networks?
- Why do you participate in a social, professional, or research network?
- What makes participation rewarding?
- Do you have a champion in your organization that helps to build relationships? Who is this person and how have they helped you?
- Who is responsible for this network? Please explain.
- How is it managed or maintained?
- How are participants identified and recruited?

**PHASE 4: OPINIONS ON BENEFITS AND CHALLENGES OF AN ONTARIO RESEARCH NETWORK IN DENTISTRY.**

**One possible resource to match clinicians and scientists interested in similar research goals is to create a network in Ontario to promote research partnerships in dentistry.**

**If such a network was available to you tomorrow. – What would you want it to look like? What are your expectations? What would you like this network to provide you with?**

Prompts

- Who would be the stakeholders in this type of clinician/researcher partnership?
- What would be your incentive to participate?
- How much time or commitment would you be willing to dedicate to these partnerships? Would you want to commit time to doing administrative duties e.g. meetings, filling out forms, being on committees, etc. Versus doing the actual research?
- How should research projects be prioritized and by whom?
- How quick would the turnaround time for the research project to be completed?

**If you were involved in design of a network, what would you recommend?**

Prompts

- Please explain how management would work.
- What types of studies should it be used for?
- Who are the participants/stakeholders that make up the research team?
- Can you say more about what this initiative would look like?
- The literature says these are helpful: funding, support, space for in-person and virtual meetings, platform to share research interests, champions for introducing partnerships, avoidance of tokenism

**Can you tell me about resources provided by the network or the stakeholders? (technological, financial, human, physical space, training, incentives, etc.) that could support this network?**

Prompts:

- Funding
- Training
- Methodological support
- Research Co-ordinating support
- Incentives to participate
- IT support
- Online profile – what are the challenges? Who will manage?

**Can you tell me about strategies (communication such as frequent meetings, project management documents, accountability, project management documenting scope and accountability, etc.) that would support this network?**

Prompts

- Frequent meetings
- Preparing project management documentation outlining scope, deliverables, timelines
- Communicating roles and expectations
- Proving an understanding of the time commitment
- Clarifying accountability
- Creating a timeline
- Following up on deadlines
- Ensuring all voices are heard/respected

**Do you have any further questions or comments that you would like to add?**

Thank you for participating in our study.
