# Supplementary material for: Exploring the divide between dental clinicians and academics for more inclusive partnerships: perspectives on building a research network
Source: BMC Oral Health. 2025 Jul 6;25:1122. doi: 10.1186/s12903-025-06437-w (PMC12232693; doi:10.1186/s12903-025-06437-w)
Supplement: Supplementary file 2 — Supplementary Material 2 [file 12903_2025_6437_MOESM2_ESM.docx]

**Additional File 2: Summary of Themes with Participant Quotes**

| Theme | Supporting Quotes |
| --- | --- |
| Across the Divide: Canadian Context | *Limited experience with partnerships*  C3: “I was a co-author on a paper recently published in the [Journal], which was looking at [topic] along with [Professor1], [Professor2] was involved as well, so we kind of followed along as they were gathering data. But I was peripheral to it. You know, they were carrying out the sort of the data collection and analysis, and that sort of thing. And then I was part of you know, writing the actual article. Similarly. I co-authored an article with [Professor] and others, based on some research that she was involved with. And again, I wasn't part of the day-to-day research or data collection or data analysis. But again, I was part of the writing the actual paper. So that's been my involvement. So it's been somewhat peripheral, mostly on the writing end of things”.  C10: “Yes [I was involved], but very peripherally. I remember sometime in the nineties somebody was doing a study. I participated. I was just a participant in that. So I was approached. I have been approached by various grad students over the years to do stuff, and you know, I can't really remember actually, but just as a participant”  A1: “the most recent one [was] the systematic review, everybody involved was in academia. I mean, I didn't know every single participant….but the people that I did know were definitely all in academia”  A4: “I go to the specifically the [study club]. Both academics and private practitioners are there…it's more like a study club kind of thing. But I think it is related. So I think that's a good forum. Sometimes, you know, I think study clubs are primarily what would be appropriate for this”  A9: “Two of my instructors actually came up with an interesting question within our teaching. And I thought it was really interesting, because I looked, it has to do with [procedure]…I looked in the literature to see if I found the answer for that question, and I couldn't find anything definitive in terms of what's the best or the most evidence based way of doing that procedure. So then, I actually said to them, we should actually do this because I didn't find this anywhere. And they loved the idea…but it stopped there. It's very recent. So it happened a few months ago. But actually, this is something that maybe at some point I might take [up]”  *Running a practice is difficult in Canada:*  C1: “[Not just a dentist], you're also a human resources person, you are also health [provider]. You do your health monitoring, you do your this, your that, and everything else. You wear all these hats. You are responsible for everything. Right?”  C11: “I don't have time, because I have a practice running. But you know the last 2-3 years, as I reflected more, there things that I am now exploring myself. One is, how to become a more effective educator… And so I am trying to see what methods of teaching are more effective. I'm always now thinking of possible projects, I don't know if you would call research projects, but I am exploring areas, and this is really uncharted territory for me with my experience.”  A7: “There is still that's sort of separation in [Canadian] dentistry between being a health care provider and being a business person. That doesn't really exist in medicine. In medicine, you are healthcare provider. So business side is not even sought, not even considered. By business side, I mean, I need to see X number of patients in my chair every day, because XYZ and so on....So there is a business side of dentistry that detracts a little bit, a lot I would say, from the innovation, from being able to spend time with the basic researcher, because there is money to be made;”  A11: “They [dental students] finish the program in a huge debt. So they have to work hard to be able to pay that, you know, like everything is super expensive in dentistry, and again, I mean, there's no financial reward in research, right? Like people do research for love, whereas there's a lot of financial reward in clinical work. So you know. I think it's really, it really depends on how interested the person is.”  *Canada is not big enough*:  C5: “Dentistry [in Canada] is not a big enough field for that, and it's just barely a big enough field for that…..and there aren't very many [specialists] in the country”.  A6: “So I think it would have to be a bigger catchment area than Ontario to get the critical mass of people that you would need to make itself sustained. Now, whether that's national or international...”  A14: “The ADEA [American Dental Education Association]…it’s not just US, it’s US and Canada…the ADEA in the US is huge, and they have incredible resources that allows them to publish and do studies. They can’t do that in Canada, We are just too small. We don’t have the resources to fund that, to pursue it. It’s a matter of size to be honest.”  *Canada versus other countries*:  A3: “What’s really interesting is the difference between Canada and probably the US, and Europe. For example, in Italy, there are so many dentists in private practice who [collaborate] with university. They have very careful defined protocols and do work in their private practice. There is an incredible number of Italian experts in [specialty]. Some are retrospective, prospective, but in this, I think somehow, in North America certainly in Canada, I am not so sure that people are willing to make the sacrifices needed to contribute to research”  A9: “They took the model, and they actually use it quite successfully in the States… A lot of research comes from private practice. Subject participants are enrolled from private practitioners…you can actually enroll people in a prospective studies with a multiple practice network, for example…But a research organization and private practitioners can collaborate the same way as an academic institution with a clinic of that institution. There's no difference, in fact, because of dentistry, you can have a larger number of subjects or participants be available from a busy practice.”  A7: “[In the UK]…so these dentists [in collaboration with academics], were practicing dentistry, they were doing private practice, doing education, being program directors, and so on…this has been one of the key drivers that does enable us, over there, that to try to maintain a good understanding between what basic researchers need and what dental professionals need. So [in Ontario] that system does not exist at all, I am not sure that there is even a desire for it. |
| Across the Divide: Perceptions of the ‘other’ | *“They” May Not Want To Collaborate*  C3: “We'd also have to find out what the appetite is from the research end of things. Maybe they don't want to. Maybe they look at it [and say] this is going to be more complicated…when we get more people involved”.  C8: “If they [academics] have an open mind and they are open to discussion, then, I find that ego is not an issue here. And that's really important in collaboration, because my experience has been that academics have an ego… [they] think they're better than the clinicians…you can’t discount the clinical knowledge that an experienced practitioner has.”  C11: “I do know that a lot of dentists who have reached out to me, and they actually feel they want to learn how to do better [specialty] treatment, you know. And so I think, clinically relevant things, like how to do things, which we call it clinical tips and tricks and pearls right? Which is lacking because there's no evidence right? And that's why there's a disconnect between the researcher and the clinician.”  A6: “The difficulty becomes I think, in our profession, is that many, many people pick the profession for lifestyle. So when they get out, they're okay to go to work, but they're not interested in doing much beyond their work hours. Those that are have already self-selected themselves into study clubs and other groups. And so, to add, one more may not be a real value”.  A7: “The system is set up as such that you will not achieve that goal [the Ontario network]. So effectively we got…on one side a bunch of research scientists or basic scientists of whatever you want to call them, said to do research and in many cases do not understand a clinical problem, and do their research in their own corner…on the other side, you’ve got a clinician…who would like to do research, but they don’t have a ton of resources or experience to do that. So it’s a very difficult equation”.  *“They” Need Training*  C2: training would be the big factor of someone who is not exposed to [research] at all. I would need to know from scratch how things work. I think the only exposure to research and writing papers and doing statistics was when I was in dental school, following that, I haven't pursued it in any way, so I’m kind of rusty at this point. So if there was any involvement, it would have to involve some sort of training to actually be able to familiarize myself with what it entails.”  C9: “But there's also a fine line, I think, between getting people to participate who are in practice in these things….How do you gauge the type of practitioner that you invite to participate? Yeah, without being mean, you know, without being mean. But how do you? How do you engage? Who is okay to participate?”  A5: “And the other problem is…when the clinicians do decide to do some research, and they're not really trained. You know, and I'm looking at them going. Oh, come on, like, where are the controls? Like, just the most basic stuff, right? But they're not trained scientists. And I'm saying, get someone you know, form a collaboration. People would love to collaborate and sort of really, make this into a good study.”  A6: “If you don't have the training in research to understand methodology. You may want to ask questions that can't be answered. You may disagree with the question, because it's not the question you want, but it's really the question that can be answered based on methodology. It's not only methodology, it’s the background evidence to know what is the context that this question is set in. I don't think you can expect that someone who's not a clinician, even if they're a well versed, driven clinician, you know, passionate about research, to be able to have access to the best quality research and the ability to analyze it and undertake managing the evidence, the way someone who works with graduate students or with researchers in a research institute with a variety of other researchers, who are all graduate degree certified, and have years of experience.”  A8: “They are amazing clinicians, but I don't think they have a research background. So it's really, how to structure right?...How do I define the number of samples?...They would have problems if they had to do it by themselves. I don't think they would have, probably they wouldn’t be able to define maybe the most appropriate methodology to answer that question. However, with a little bit of background that I have. When I suggest, how about this, this, and that? They go, Yeah. Exactly. That's what we want to, you know. So they add up, it's like an amazing combination. It's really maybe the background [is a challenge].”  A9: “I think it would be useful for people that are practitioners to be knowledgeable of what our typical requirements to answer good clinical questions, so that they are by default keeping those measures…a lot of times, people have large numbers of subjects, but they have not recorded things because it's not profitable, or it's not in the interest of the patient. It's not a research study, patient is there for treatment and therefore patient doesn't necessarily get all the recordings done the same way that can lead to answering the clinical question in a valid scientific way. Because that data doesn't exist. The patient exists. There are some data that exist, but there are too many missing elements of data...So yes, what happens is that undergraduates feel that a lot of this stuff they will never need to use. But then, 30 years later, the same person has now become ‘I wish I had done this. I've been doing this all the time, but I don't have the data”  A11: “And again, it also has to do with exposure. Like a lot of students, they go through the entire 4 years of dental school without having absolutely no exposure to research, or very little exposure to research. So when they come to us to become residents, they have absolutely no idea of how to even read a paper, they don't even know if it is a good paper or bad paper, because they had absolutely no understanding or training on that.” |
| Across the Divide: Perceived lack of respect | C8: “Unfortunately, it's undermined [clinical research]. Inadvertently, I'm not saying it's deliberate. But those who are reviewing our work. They're not seeing how relevant it is to the public, to our patients. They're not seeing the relevance of answering these clinical questions….It is so important that people recognize clinical research…so where is the clinical research being done? And we don't have the money and neither do we have the resources to apply for a grant”  C12: “The second one [challenge] was to find someone at the faculty who would help us…[removed] a lot of the people here weren't that helpful. So getting a professor who would work with us and understand what we were doing, so many of them were so dismissive. So that was a huge discouragement. I found the [department] was really standoffish and completely not helpful when I would have thought they would be.”  A3: “You have very little clinical research at all, [removed], and we don't have any support. For example, in the biological research, of course, there's a lot of funding from government and other agencies. But the faculty is also very supportive with personnel. In clinical research, No”  A7: “And I think we're using pace very fast because we still rely on that all old guard to be spearheading, and in some sense they are, but effectively the old guard is kind of holding back next generation coming up, and therefore we cannot try to drive those relationships.”  A9: ““[lack of collaboration] reflects the genuine lack of respect amongst those that have chosen to work more towards one side of the fence. Whereas there should be no fence here. But you know the fence exists and people think of themselves as either highly academically oriented, therefore more proficient…than those that are more in clinical, or seem to be at the other side of the fence, and that fence needs to be broken down”  A10: “This goes back from, I graduated in [year]. Basic science was king and still is. And you know, basic science is important for any medical, biomedical endeavors. But I would say that clinicians have never been properly supported to do research. But you know, that is not something [you see] in medicine. Half the doctors at [hospital] are clinician scientists....So to be succinct. I don't think there's the support for clinician researchers in dentistry overall. And I think, because basic science research has been predominant.”  A11: “They [academics] would say they would feel like, you know. You don't know science. You don't know how to do science, you're just a clinician, you know.”  A13: “So it's like an old boys club which is very difficult to penetrate. They play among themselves. They don't want to involve any new ideas….So, having an open platform circulating, who wants to participate in this, you know, come forward....[Currently, it is], this is open, now [it’s] closed, because we already filled the quorum with 5 of our good friends who [have] already been working on this. That's what has been happening, and that's not a good thing. What happens once they retire?...How will we get the legion of good data? And it has to be like, it's an open platform, not on a closed group of people deciding on who to be in their study, who is going to contribute? Who's going to get the money? It all boils down to that” |
| Across the Divide: Physical and Social Barriers to Collaboration | *Physical Space:*  C3: “Because I see a lot of people in the elevator, and I see them press the button for the [research area], and I have no idea who they are. Now you meet some of them at [event for research], which is something nice… where we bring the research into the mainstream where it's actually oh, people in the rest of the building can go see what's going on. But it's not that common”.  A5: “So I know there's a lot of clinicians here, in our faculty, who really just work in the clinics. They're on their feet all day, they don't leave the [clinical area]. They've never come up. I'll invite them up to the [research area] and they go “There's a [research area]?” I'm going, yeah, there's a [research area], that's where all the research is, you’ve never been? So yeah, there is really [this divide].”  A9: “You have to get to people, and how do you get to them? Sending them stuff in writing doesn't make any difference at all. [I] skim the Faculty Newsletter, when it comes. But I would say most people in private practice don't.”  A11: “You know the researchers were always like on the [research area], isolated from everybody else, whereas the clinicians, and it's I always felt like researchers used to look down clinicians and vice versa, and they would never integrate, and they were never comfortable together.”  *Social Media*  C3: “I would say that I'm really not a social media person. I'm not on Twitter. I'm not on Facebook or Instagram, or any of those other platforms. In part, because I don't have time, between just being on the computer doing my day-to-day stuff, as well as texts, emails and phone calls, that's almost enough. And once I'm off duty, I don't want to be back on the computer. And somebody asked me recently, are you on LinkedIn? I said, no”  A9: “But they [academics presenting at conferences] get a lot of questions from the audience on [social platform]. So people will reach out after the conference, or after the lectures given to these individuals. If people have gone on social media, some people are so busy with this that they actually say that don't send me an email.”  *Communication/Research Dissemination*  C3: “You think about all the research that goes on, [removed], but let's say all the research that goes on, how many of those projects have to come to fruition with a clinical application? We're now using this….There’s an awful lot of research….and then you think, well, where are these products? What happened?”  C8: “Where is the repository of work that's being done? How do we know who to collaborate with? If I want to, if I want to even develop ideas with an endpoint, you know, it's nice to do an app project. Yes, but what relevance does it have to clinical dentistry? Right? So that's where I come in. But I need people who are open to promoting their research. So I can develop that collaboration. So that's a big stumbling block.”  C9: “I know that they send us emails. I get emails all the time for research rounds or this or that…they [are at] very inconvenient times. It's usually your lunch hour, and I'm not even at the faculty, I'm seeing patients, which is not my lunch hour there. So you know, maybe providing a little bit more variety on the times that they're available. Would capture different people and not the same people all the time.”  A5: “We are searching for effective ways to get the clinicians and the basic scientists together to get, you know, the clinics and the [research area] together. One of the models that was suggested. But we've never done was to have you know, research rounds.” |
| Bridging the Divide: New Collaborative Research Network | *New Network:*  C1: “I think it's a great idea. I think you have some logistical hurdles to go through, but they're not insurmountable. I think they're totally doable. And I think, for the dental community, it would be really good. It brings us together just a little bit more, and gets everybody interested”  C2: “Yeah, it's a good idea to integrating clinicians along with research, because it's very hard sometimes to solely focus on the evidence, because evidence may not always apply to every patient you see in a clinical scenario…So I think getting clinicians integrated into the research and finding out stuff that reflects more what is in regular practice. I think that's very important. So I think it's something that's important. And I think it should be given a chance”  C3: “Part one would be somebody in research has got an idea that we want to look into this, whether or not they wanted to seek out some outside individuals to be part of it in some manner. The second part of it would be those outside saying, I'd love to see some research on “this”. So I think there's two elements there”  C4: “I think that this is a great start. The fact that this, even, you know, is being discussed, and you're really looking at the research. I think that's really great. I think the challenge now will be, finding private practitioners with right the time, and, of course, can be a lot of volunteer hours to start. But I think starting even starting a group like an actual group, not a Facebook group, or any of those social things, but like an actual association, an actual group, an actual something. I'm not sure what, where you have true, true, passionate people who feel that this is an important cause, and you know, would put the effort into it just because they want to do that, not because of any money, or because of any incentives or like whatever right? It's genuine, you know.”  C6: “I think that probably the single biggest barrier is just not knowing how to take the first step and get involved. I think, there's a lot of great academics and researchers who can be sort of the brains of these projects. I'm happy to be a worker bee, collect data…I guess, not knowing the first step to take to say, Okay, hey, we're interested. These are the resources we have that we're willing to share with the academic community. How can we help”  C7: “So you share ideas. You share information between dentist and between academics and dentist. so they may explore something that I don't know. So networks are really important for dentists.”  C8: “But as the direct answer to your question. You know there's no official collaborative network, but I have tried to make the best of my situation and find people who complement what I do”.  C10: “I would really love to collect all the data from my office to show what the situation on the ground actually was….The dentist who works in their little office, in you know remote rural area of the province. We'll talk about the province. Somebody who's north of Sudbury, or, west of London and east of Kingston…those people would have actually an absolute gold mine of data. Because they tend to stay in the same practice and have treated the same families of patients over generations. So that's a tremendous amount. So you need somebody to identify the characteristics of those people and what it is that would interest them about getting involved in something like this. So you're looking at sort of an older population, too. For some of that stuff. An older population of practitioners who tend to be more philanthropic. And as they go on in their dental practice lives, their dental careers, they tend to get a little bit more, sentimental is too strong a word, but they tend to appreciate, really, all the things that dentistry has given them and do want to give back. So you have to identify that altruistic motive.”  C11: “That's when I kind of I feel I am not quite sure where to go, I reach out to people casually, I need to have a meeting. But between the lack of time and everyone so busy with so many of their own things, it does become a barrier to get things moving...I think it would be helpful for me. It would be like, okay, I need this, who should I contact right?.. that's where I feel like I'm stuck in some ways, because I'd like to do more, but I don't know how, I need a little bit more guidance…I feel like I'm intruding on their [academic’s] own time asking too many things since they're so busy. Then I don't ask, because I feel like…I'll ask another time. So one thing trickles to the other. And so you I feel that, just the progress will be very slow if I want to move things quicker, I think a network would help me get things moving faster.”  A3: “But, you know, I'm just thinking of this now. Because, I mean, who would ever think that [university], would produce clinical research in private practice? Never happens. Because there's no way to set it up. There's no funding to approach. I mean, there's funding from American institutions like the [professional academy1] and the [professional academy 2] and other groups, that can provide some money, if you have a good application…if you put together a really good protocol. And you said, Okay, well, we have these 3 practitioners. They're all trained. They're all basically prepared to do this.”  A4: “Because, you know, even if you think about it, even within the building, we have major difficulties connecting basic scientists and clinical scientists, right? In the same building. So, to reach out to all the dentist you would need one extremely, I mean. I think it has to be grassroots, in the sense, that you would need to have one extremely enthusiastic person who's life calling is to connect general dentist to academics, and kick start research projects”  A7: “But having a clinic full of private patients, you can run so many clinical trials, yet we aren't doing any. And the reason why we aren't doing any, is because, again the system…hasn't created a clinical trial unit…we have all the right people but they just need to be brought together, and that has hasn't been done, because again, there is “It's not what we used to do in the past” because there is a notion of archaic research approach.”  A8: “I just want to say that I loved this interview. Thank you so much. And I also thank you because this is so much needed. You know, we need to be, we [are] struggling with all this clinical research and how we gonna do this or that, we have to be creative about it, right? And you know, and I think that involving clinicians, because I have my feet in both worlds, I can tell you that this marriage can only be successful if we [are] able to really bring both sides together.”  A8: “I love this idea. I think it's about time. I wish this can happen. This is a gift right for both of us [clinicians and academics], because you know what, we sometimes, we take clinicians for granted. But they are really very busy. And at the end they don't have much access to this kind of resources. As we just said, they just simply don't know, or don't get exposed to that, because that's how their profession runs, right, in many cases. But I would, but I would argue that many of them would be very interested if they had the opportunity  Participants perceived an optimistic view that it is best to find clinicians and academics who are passionate about research and would not require a monetary incentives to participate. The word “grassroots” was used by several members to describe the types of individuals who would be willing to start the network or at least be involved in the beginning of the network with none, or little, monetary incentives.  A9: “Yeah, [the American clinicians] have a collaboration [with academics] so that a proper research ethics board application can be made to look into the data....a lot of research has been done in the States. There are numerous examples of this, even within [specialty]. A lot of research comes from private practice. Subject participants are enrolled from private practitioners.... Everything. There should be no reason why of collaborative practice network cannot be affiliated with a university setting or a researcher setting wherever regardless of whether it's university or not university. But a research organization and private practitioners can collaborate the same way as an academic institution with a clinic of that institution. There's no difference, in fact, because of dentistry, you can have a larger number of subjects or participants be available from a busy practice.”  A10: “But you know my friends are, general dentists, and so on, and I don't think they feel a connection whatsoever with research type of thing. but I think there is a need to bring those kinds of people in and, like to help develop research questions. To help shape the project and then, you know, I think there would be instances when a research network would be important.”  A11: “We don't do enough clinical research. I feel we're so behind compared to medicine. You know. They're so strong in medicine, you know we will tell ourselves that we are clinician scientists. But to be honest with you, like, I feel like we're either clinicians or we're scientists, you know. That integration between like being a full clinician side. I don't really think none of us is, you know, because we don't have this collaboration. So yeah, so that that's like, I have a really strong opinion about this, because, like I said, I've always wanted to get this together”  A14: “Should we pursue it? Yes, we should. But I think, expect some frustrations at the beginning that it might be hard to get take off on this. I would look at the US Model, see how that's working in the US. Because they are doing it and see, maybe learn from them and see, if we can model something in Canada to do the same thing? But I mean there's a lot of untapped potential out there.”  *Decision-Makers/Champions to Start a Network:*  *Clinicians*  C6: “But you also need to have good connections with the front-line clinicians. I think that it's collecting the private practice dentist and getting them involved can make the experience richer”  C10: “The dentist who works in their little office, and remote rural area of the province. We'll talk about the province. Somebody who's north of Sudbury. Or, you know, west of London and east of Kingston….those people would have actually an absolute gold mine of data. Because they tend to stay in the same practice and have treated the same families of patients over generations. So that's a tremendous amount. So you need somebody to identify the characteristics of those people and what it is that would interest them about getting involved in something like this. So you're looking at sort of an older population, too. For some of that stuff. An older population of practitioners who tend to be more philanthropic. And as they go on in their dental practice lives, their dental careers, they tend to get a little bit more, sentimental is too strong a word, but they tend to appreciate, really, all the things that dentistry has given them and do want to give back. So you have to identify that altruistic motive.”  A2: “the clinicians at the front lines”  A7: “So, for start there needs to be a consensus, a willingness on both sides to try to be wanting to work together. If you don't have such, there's just no point to try to bring people at the table, because they are simply not gonna talk to you, they will say ‘you’re wasting my time’”  A8: “I think that the idea is amazing. I think that it would be amazing if we could build a network in Ontario. This would ideally, we would want to, I think, involve all specialties in dentistry”  *Academics*  C5: “I think you need to talk to academia and people who are into research, and, you know, consistently into research.”  C6: “Yeah, it's a great question. I think it's a very compelling idea. I think that you need to have a combination of your academic minds who understand research methodology and can ensure that the research is conducted on sound scientific principles, and you know we'll have good internal and external validity…”  C11: “But to be honest with you, I think this is something that should come from us [dental school faculty], like we are the people who should be generating knowledge.”  C12: “First off would be the Dean, the Dean of our institution would be really instrumental, and then the Vice Dean of Research, to me those are the key players to get that going”  A13: “So there's a new concept called pragmatic trials, which means, as I said, as long as there's certain check marks that we consider, it [data] can be pulled in and make sense, because the larger the data, the better it is. So in our case. I really feel it has to be like a big school program directors because they have the access to data…Having them involved, because you can also set certain norms when they are treating the patients, I'm just talking about like treating patients and outcomes”  A14: “To be honest, I can't think of a mechanism for clinicians, because it'll depend on the type of study….I think it would be best for the specific PI to say, this is what we need, this [is] how you have to do it. You can't break the code on whatever, and to get consent, you have to do this. I think it's just an individual let's spend a couple of hours going over, this is how you have to do it. But to me the academic has to take the lead. And it's not that the clinicians but the academic, I think, has to take the lead on that.”  *Parent/Caregivers Groups*  A2: “patients obviously are, if it's patient-related research.”  A6: “Once again it would be advocacy groups and parent groups to know about what their options are”  A9: “But ultimately they [patients] represent the question that was asked pertaining to that subject matter, which they are the representatives of, because they are the participants in that. So ultimately the focus is them, and the findings are targeting towards them as well. So they're very important participants in that.”  A10: “I think the place to start is to involve ordinary dentists, clinicians. and patients in developing the question. You know it all begins with the research question, and there's no point, studying an endodontic whatever, if it doesn't make a difference on the health of patients, you know…. But I just wanna emphasize that bringing people in at the beginning of the planning process, you know, like that doesn't take too much of a practicing dentist's time. You know a meeting at lunch or a meeting after hours. That they can do, and to get those ideas is really important. And I can't stress enough, patient involvement, too.”  A12: “Yeah, it'd be really interesting especially if it's some kind of survey or some sort of interaction with the public at large…it was like a lot of these organizations that I belong to have a public member, right? Because we all think ‘dentist dentist dentist’, and then the public member comes up with these unbelievable things. And you're like Whoa! I never thought of that...so they have a lot of good input. It's important to get a good public member”  *Government funding*  A8: “There's Federal funding agencies like CIHR. I don't know if they would be, if they would have anything. But it is ultimately clinical research, right? So they might be, because I know that they are, I'm not honestly not that familiar with CIHR. But I am sure that they do have interest in testing populations.. So I think something to explore over there. oh, yeah, Federal funding should be explored and industry funding should be explored”  C8: “As to who the stakeholders are, they'll be stakeholders in the government we need financing. Funding is a big part of this. We cannot do anything without funding our projects so small.”  *Private Funding*  C1: “You can always reach out to a manufacturer who can make this particular product and say, Hey, we got this product, of course patents might be involved. But anyway, we got this product. We think it's gonna work. We're trying it out in a clinical setting first. If this is comes to fruition. Would you like to have first crack at manufacturing it?...there has to be something in it for them”  C5: “I think you need to have the money people there….so therefore, private venture capital.”  C10: “You might have a private foundation that has an interest in oral health. So if you could identify a large donor through the Advancement Office that would be interested in funding something like that. But then this, this project would be competing with all of the other philanthropy needs of the faculties. I hesitate to say this, but you probably could get funding through some of the large dental manufacturers and suppliers. But it would have to be very arm's length, and it would have to be completely clear that none of the information that came out of this project could be used for any commercial or reputational benefit of the manufacturers or suppliers. So I don't see why they would want to fund it in that case, but they would be a source of money.”  A3: “And there's also industry, too. But industry, you know, that's a that's a whole different thing, and it's better sometimes not to be involved with industry because then there's a risk of conflict of interest, and so on, and so forth.”  A8: “I think we would have to explore other types of [financial support], maybe industry…depending on what we are testing or evaluating right? If it's extremely translational… Oh I think an MOU [Memorandum of Understanding] has to be very strong...because at the end…the data belongs to both, and a huge agreement has to be obviously established, so that the data is protected.”  A12: “I'm gonna throw it out there, there is some of the leaders in industry as well…these are all companies that are interested, they have research arms, and they want to do clinical studies of their products and new products and things like that. So that's where they test right? Like, that's really important.”  A13: “So the sponsors can like, you know, maybe help us book the venue [for seminars] because the venue itself is a big money, and then when you go there. You do want to provide some refreshments. So like I said, the good part of dentistry is like they have quite a bit of industry involved. They want to showcase their products. So finding sponsors is kind of a very good idea.”  *Professional Associations*  C3: “Yeah, well, I would say think of the ODA more as not the host, but really the conduit that would connect people to the faculty.”  C12: “Well, I hate to say it, but you're probably looking at organized dentistry because that's, I mean, the majority of clinicians probably belong to one or more groups of organized dentistry groups. So that's probably the best place because if you focus at the faculty, you have a very skewed set because most people here, many people here aren't in private practice. Many people here are retired and many people here are academic researchers. But if you want to get out the clinicians who are in the trenches, day after day doing dentistry. I think it's probably like a mail out. I know in the past they've done mail outs to every dentist in the province through the RCDSO listing. Uh questionnaires or, you know, calling people up through the ODA, and one of their, you know, social media forums or something. Those are the two best ways.”  A6: “Well, I think certainly the Professional Associations. Whether that's the Canadian Dental Association or the provincial organizations, because if it if this comes from the universities, I'm not sure it would be that welcomed among the community, not because of animosity. It's just like, well, what do I have in doing a bunch of university research.”  A14: “So yes, I think that [professional associations] could be a vehicle if there was interest in doing that. I think that would be great. It's often not the primary objective of associations. It depends who you're talking about. If it's let's say Canadian Dental Association or the Ontario Dental Association, that's not their prime mission, although they do want to enhance oral health care. So when they have that understanding, they may feel, yes, there's a role for this, because this will advance the profession. But it's not their primary objective. So it might be more of a challenge to get them on side for that.”  *Champions in organized dentistry*  A1: “So you know, I have known several dentists who have been involved in the ODA and CDA. And I would say that those are the kinds of people that would be very useful and helpful and getting feedback from, and even if there were a project that were to be wanting to get started. These would be the kind of people that you you'd want to be involved from the beginning, because they already have an expressed interest in this kind of the project.”  A6: “My guess. I could be wrong. I think it would have to come from dentists who have an active role in organizing dentists who see the value in collaboration, of course, with the universities”  C1: “So if the clinicians or the researchers were to hook up with the ODA clinicians I think they'll have a nice broad base to help them with their studies, or to have that interaction. And I think, and then by appealing to them, then the clinicians can say, Yeah, okay, I'll try that. And then there can be some feedback or some, you know, communication back and forth as to how everything works. And hopefully problem solved.” |
| Bridging the Divide: Using Existing Channels for Collaborations | *Existing Networks:*  A6: “So, there was a core group of people who had done research in that area, that sort of tried to expand the scope, and in doing so, they just invited people who might be interested to come, but also identified individuals that might be of value…and then started out down that path with people being invited to participate at different levels, as well as people who wanted to volunteer to work on research projects or other projects”.  A8: “I've been invited to speak to groups of clinicians. That doesn't happen that often. I'm going to speak to one of the study groups…Because then I'm explaining a little bit of my research and also the translation. Right? So these are interesting interactions that may happen”.  *Professional Associations as Communication tool, not network*:  C1: “The ODA has a large base of dentists. Basically, you know, addresses, emails, whatever. So you can always hook up with them, because they pretty well go throughout Ontario to get your clinics that may come on board. So if the clinicians or the researchers were to hook up with the ODA clinicians I think they'll have a nice broad base to help them with their studies, or to have that interaction.”  A2: “We've had recent discussions in our specialty organization about whether we need to enhance this link between private practice, ideas or questions, and whether like research should be done at the masters of science level to help to collate or to bring some sort of understanding to what is observed in clinical context. This, I would say, this would be an informal conversation between a few members of our community of our networks. So it's interesting that it's maybe just the beginnings of conversations.”  A4: “I mean the ODA it came to my mind first, because they are the ones who practically catalogue all the dentist in Ontario. Right? You can get around that and but there is also, I know there's one called Women in dentistry, which is, I think, it's more of a female oriented organization, but I don't know who they actually encompass, or who the members are. I know they have all some mixed academics and private practitioners, so that might be the forum as well... But I think the ODA or the or the RCDSO. So you know, because they also have a register of everyone, just for reaching out and trying to kick, start something.” |
| Bridging the divide: Support for clinical research needed | C8: “The faculty of medicine, I think, is, they have much more advanced and intricate networks that we can derive information from, from us in dentistry, we are just at the first rung of the ladder. We have a lot to learn”  C10: “Right…most dentists are self-employed. So they do not get sick pay. They do not get vacation pay. They do not get stipends for continuing education. They don't have pension plans. They have a very expensive staff that they have to support and do all the contributions. So dentists are not eligible for EI, women dentists are not eligible for mat leave. There's no compensation for mat leave. So every hour a dentist takes out of their practice is taking their livelihood away from them. It's actually costing them money out of practice like out of pocket. So every hour you expect them to be in a meeting is taking away their time where they're working to pay their bills in their practice and have something left over. So I'm not, you know, not playing a violin for dentists. It's a great profession, and if you work hard we'll get through all of this. But it's a very different mindset than somebody who was employed in the hospital.”  A8: “The second thing [challenge to collaboration] is always infrastructure and finances. Right? I mean you, you have to pay, if you have to pay for a summer student, even if it's a summer student, a 3-4 month research program. It has expenses you have to pay for, you have to buy materials, you have to pay for the equipment that we're gonna use to analyze data, to test materials, to test samples. There's always some cost involved. I don't, you know. I don't think that, unless you have it ready. So where the resources are gonna come from, right? I think that is a big thing to prevent many people to get involved.”  A10: “In medicine, you know, half the doctors at [hospital] are clinician scientists. You know they have done PhDs, they're supported in their clinical research endeavors…graduate students, to get their specialty license, have to do research”  A11: “We don't do enough clinical research. I feel we're so behind compared to medicine. You know. They're so strong in medicine, we will tell ourselves that we are clinician scientists. But to be honest with you, I feel like we're either clinicians or we're scientists. That integration between like being a full clinician side. I don't really think none of us is, because we don't have this collaboration. So yeah, so that's like, I have a really strong opinion about this, because I've always wanted to get this together”  A13: “We see [Greater Toronto Area Hospitals]. They all are collaborating. Let's do something better. Why, not in dentistry right? So I think that barrier has to be lifted….the network has to involve senior mid-career, early career researchers as well as clinicians who can see, again, there has to be senior who can guide, there has to be mid-career who can work, and there has to be early career who has to aspire to be in that position. So it has to be a good cohort of people, like-minded people. You cannot have everyone in the same platform. But you can start somewhere.”  A13: “The private practitioners, I feel whenever we talk, they do have ideas, because, you know, they want something hands-on right? Maybe not exactly biology based. But technique-based because they do that day in, day out. I do that one fifth of my work time. They do 100 of their work time like absolutely, they are much more well versed so, but the only issue is like they don't have access to what to do next. Like a lot of people have ideas, but the bad part is, there's a roadblock. Who do they approach? That's kind of a tricky. And at the same time, if you don't have a proper path. The idea dies off.” |
| Bridging the Divide: Administrative and funding support for clinical research | *Administrator/Research Coordinator*:  C1: “In that you have one team leader that connects between the researchers and the clinicians, right? And if there are any questions, other than on the meeting…that they can email them [to the person]. And then any let's say, any changes or solutions…everybody can get a broad email sent to them that says we're going to change this product, this procedure…So keep everybody in the loop keep everybody informed, but keep it simple”.  C2: “I think the biggest thing in this, would be trying to coordinate [it], because there are so many multiple bits and pieces and numerous individuals involved in such a research project. I think coordination of everyone [so] each one can do the time, the stuff in their own time, but having to coordinate everything to get something together, it's got to be some, but somebody, or at least a group, to be able to get everything together. I think that is very important. Even if everything is in place. If you're not able to get everything together. I guess it would take longer than it needs to if it's not actually compiled.”  C10: “You'd have to hire somebody. The network would have to hire somebody. You couldn't get a volunteer to do that. If you want to involve dentists in private clinical practice, you're going to have to make it as easy as checking off 10 boxes, in the answers to whatever it is I think you have to basically make it as accessible and as the least time consuming as possible”  A2: “I would say, human resources, and that goes along with funding for that human resource to be that central coordinator to facilitate the collaboration. That's always difficult to find time, or like carved out specific time for these conversations to happen and also the time to prepare for the meetings, right? So establishing some sort of process, so that when meetings occur it becomes very efficient in those points of meeting. Yeah, I would say, like administrative support......I really think there needs to be a centralized person, whether that be a member of the research team or whether that be an additional supportive member as an administrative assistant to help coordinate, because that does take a lot of time, and some of the micro tasks required to achieve the collaboration.”  A3: “So and I mean the person has to be properly compensated, right? It's not going to be like $20,000 a year. It has to be something that's appealing to someone as a career, as a career move, but that involves benefits and vacation pay and sick days, and all this kind of stuff, and it's not so easy to find such a person. But if that person could be found, that would be a huge step towards undertaking a project like we've been discussing, with private practitioners”.  A5: “That concept [clinical research coordinator] obviously didn't exist back then. We were all just struggling, and really what you needed, was a professional clinical coordinator who goes “I'm dealing with this, I'm managing the labeling, shipping, the distribution. I’ve got it. If you have questions, you come to me, because I'm the central person. I know what all the labels, all the numbers, all the patients. I've got that information”.  A6”…administrative people that should be helping us or could be helping us, to write grants. [Because] a lot of the times. We have the ideas. We just don't have time to sit down to write the grants, right? So, you know, like, have a like a research manager or somebody who would be facilitating all of this....So we need somebody to negotiate and advocate on our behalf to say, ‘Hey, this is ridiculous, Let's get this project started’. Otherwise we're gonna lose this money, you know, and that's what we need, and we don't have. And to be honest, which is sad...let me backtrack. We do have some [administrative support]. Yeah, but we're way behind. We're way behind with all we need. Right?”  C10: “It would have to be funded by the Government.”  C12: “I think it would be nice to have funding at the Federal level for sure. Definitely.”  A6: “Well. funding is always a problem. and that's one of the advantages of large groups that involved more than clinicians is that you can get. If you can get members of industry and advocacy groups involved. They are happy to fund things of interest that may solve their problems” |
| Bridging the Divide: Commitment and Incentives | *Time and commitment*:  C1: “The whole idea is, if you want people to participate, you gotta keep it simple. Because…dentists are very busy, and, unfortunately for us, there are so many things that get ladled into our lap. [After hours] you no longer just a dentist, and then you go home, and then you’re the chief cook and bottle washer”  C4: “But if I knew that from October to February I was going to be starting a project, and I needed to have you know, Thursdays to do that, or I needed Thursday afternoon to do that. Then, I think that's more doable than say for the next year, whatever next year it's going to be, your meeting once a month, or once every couple of weeks. I think if we had a set period of time when...this is sort of where you were gonna meet. And you had some planning. you had 6 months. It's a 3 to 6 months advance notice, like this is when we're going to start this. I think that would make the make the scheduling just, and logistics a little bit easier. at least for me…. for someone like me, I would be involved until it's over. I'm committed for sure, you know. Yeah, exactly. You start a project and you finish the project.”  C6: “So at the beginning, I understand that there could be times where it's very intense. I could tell you, I could probably give you a pretty accurate answer for a lot of people in private practice…On an average work day, where you can kind of sneak in thinking about this and answering emails, it'll be something that's largely done on evenings and weekends. Because practice life is kind of like all consuming when you're there. If you have kind of like an admin day, like I try to take. I've basically made one weekday now where it's just get admin stuff done. That's kind of your flex time, where certain times you could devote, you know, 2 to 3 hours in that week to a project...whereas, other times you'd be, you can barely even get your bills paid. So I think that the implementation and integration of the clinical part would have to be very smooth… I'm happy to be a worker bee, collect data”  C7: “Yeah, I mean, maybe, according to now…I think I can dedicate one day a week, which is good. We are talking about weekdays. Yeah, one day a week, like a full day. So that's would be good for me.”  A4: “This will sound wrong. So I'm not saying that academics are busier than private practitioners. But I think academics have the disadvantage of having more on their plates in the sense that you know, when you close the door, you are still not rid of your work…I know this because I also work in private practice…”  A11: “like one of them [challenge] is like everybody's really busy, you know, like with their own, with their own projects, or like a researchers, are always busy writing their grants and getting funds for their labs and doing whatever they need. Clinicians are always busy with patients, and you know it's a busy schedule clinic and private practice and everything. So I think that's one of the biggest barriers. So you really need to be willing to do this and carve the time to be able to do this”  A12: “I mean in all honesty this place keeps me pretty busy, but I have had ideas and approached a few people in the past, but nothing's ever really come to fruition. Most of it involves sort of my thoughts. Things that I thought about are just using clinical data that we have through [record system]”  *Incentives:* Continuing Education Points  C2: “Probably continuing education points.”  C11: “Incentives, I think, as a clinician, they are looking for CEs. Yeah. you know, if they can get CEs…”  A13: “the biggest incentive is continuing education points, because all of us need the CE points”  Incentives: Authorship  C3: “I think maybe the opportunity to be one of the authors on an article. They might say, I would love to see my name out there.”  Incentives: Knowledge acquisition  C1: “You can then also offer them, let's say [university] holds courses, you could offer them a free course for doing it…and that is, that is incentive right there. So if you get a chance to, you know, see one of [Professor’s] lectures, or some who whoever else is giving these lectures that you have to that year. That would be, I think, a little bit of a good incentive.”  C2: “probably meeting together in some good place to discuss stuff. It will give us time off from the regular hum drum”  A1: “knowledge acquisition. I remembered I used to go to our demonstrators on a fairly regular basis to talk to them about things that I thought were relevant, that they should know. So if there was an update in a particular area, or a change in dental industry and I wanted to explain it to them. I would go out during the clinics and just sit down with them and go over what I thought they should know. And then, of course, there were staff meetings, I remember one of our better demonstrators saying to me, well, this is one of the reasons I teach at school. It's because I want to be kept abreast of things like this. So I think knowledge acquisition would be a big one.” Incentives: Promotion of private practice C7: incentives will be, maybe, what will it add to their practice? Second, I will say, what will it add to their experience? Like as dentist, are they going improve themselves?  A3: “The clinicians in private practice, of course, would be, there could be some benefit to them, I suppose, that their patients think that they're connected to university, and that they do some research, which is evidence-based…” *Incentives: Monetary* A2: “I guess you could do what's expected of compensation per hour, if there was an actual time logs to track the hours of commitment, and whether that be similar to their hourly expectation for a private practice clinician, would be very reasonable and motivating.”  A3: “Because the department, certainly myself, and [Professor] and other people in the department, would be thrilled to be involved with no compensation other than their usual compensation, and excited, you know, to take this this new step.” *No Incentives needed*: A4: “But you know, for academics. I don't think you would have to find a lot of incentive, in a sense, because academics are always looking for a new project. They are always looking for, you know, hopefully, clinically relevant topics. And if somebody comes to them, bit something clinically relevant, then hopefully, they would be interested to pursue it.”  A5: “I don't think you really have to incentivize it. If you went into research, you are just curious. I mean, if you're not curious, you can’t get this job. So, I think almost all scientists have this natural curiosity that drives them. If you come up with them, or if they come up with novel ideas, or if clinicians sort of go, and scientists love this kind of stuff. If a clinicians came up to you went. you know I've got this issue with [topic], that I just can't figure out” *Incentive: Validation of research* A5: “We’re scientists, we all want to see our work being translated into something practical. I mean that would be the gold standard for everyone to see your research actually become help in clinical practices. So I don't think you need to incentivize researchers, but they need money to do it. I mean, they'll be naturally curious, and they'll love to talk about it. But at the end of the day, you can't ask them to do it without giving them money, because, the resources have already been allocated to their own projects.”  A8: “I like to see my ideas. Well, you know, I want to see, it's a validation of my idea, it is the ultimate validation of my idea. Right? If I develop a material or I test a technique, or I've come up with an idea in [topic] which is such a clinical field in dentistry, right? Practical, like clinical field, and I have clinicians, you know, using and saying, this really works, you know. For me, that's the ultimate validation. That's more to me. Well, it's more valuable than actually a randomized clinical research right? Cause that's really real life.”  A9: “And so there's a commercial interest at times, or validation interest that people may have. But I think if the process existed much more frequently, we would have a much more open area of communication, and more answers can be really sought for very good and meaningful questions.” |
| Bridging the Divide: Expanding knowledge of dentistry | See Table 2. |
